# Age related differences in individual quality of life domains in youth with type 1 diabetes

**DOI:** 10.1186/1477-7525-2-54

**Published:** 2004-09-22

**Authors:** Julie A Wagner, Gina Abbott, Syretta Lett

**Affiliations:** 1 Department of Behavioral Sciences and Community Health, University of Connecticut Health Center, 263 Farmington Avenue, Farmington, CT 06030, USA

**Keywords:** diabetes, children, adolescents, quality of life

## Abstract

**Background:**

Investigating individual, as opposed to predetermined, quality of life domains may yield important information about quality of life. This study investigated the individual quality of life domains nominated by youth with type 1 diabetes.

**Methods:**

Eighty young people attending a diabetes summer camp completed the Schedule for the Evaluation of Individual Quality of Life-Direct Weighting interview, which allows respondents to nominate and evaluate their own quality of life domains.

**Results:**

The most frequently nominated life domains were 'family', 'friends', 'diabetes', 'school', and 'health' respectively; ranked in terms of importance, domains were 'religion', 'family', 'diabetes', 'health', and 'the golden rule'; ranked in order of satisfaction, domains were 'camp', 'religion', 'pets', and 'family' and 'a special person' were tied for fifth. Respondent age was significantly positively associated with the importance of 'friends', and a significantly negatively associated with the importance of 'family'. Nearly all respondents nominated a quality of life domain relating to physical status, however, the specific physical status domain and the rationale for its nomination varied. Some respondents nominated 'diabetes' as a domain and emphasized diabetes 'self-care behaviors' in order to avoid negative health consequences such as hospitalization. Other respondents nominated 'health' and focused more generally on 'living well with diabetes'. In an ANOVA with physical status domain as the independent variable and age as the dependent variable, participants who nominated 'diabetes' were younger (M = 12.9 years) than those who nominated 'health' (M = 15.9 years). In a second ANOVA, with rationale for nomination the physical status domain as the independent variable, and age as the dependent variable, those who emphasized 'self care behaviors' were younger (M = 11.8 years) than those who emphasized 'living well with diabetes' (M = 14.6 years). These differences are discussed in terms of cognitive development and in relation to the decline in self-care and glycemic control often observed during adolescence.

**Conclusions:**

Respondents nominated many non-diabetes life domains, underscoring that QOL is multidimensional. Subtle changes in conceptualization of diabetes and health with increasing age may reflect cognitive development or disease adjustment, and speak to the need for special attention to adolescents. Understanding individual quality of life domains can help clinicians motivate their young patients with diabetes for self-care. Future research should employ a larger, more diverse sample, and use longitudinal designs.

## Background

Quality of life (QOL) is now recognized as an important outcome for people with diabetes. In general, diabetes has been shown to negatively impact QOL [1]. Tighter glycemic control is associated with better QOL, despite the increased treatment demands it commonly requires [2]. As standards for optimal glycemic control get more rigorous, and as medical treatments for diabetes develop, a better understanding of the personal meaning of disease and related QOL would be beneficial.

The measurement of QOL is evolving, and a state of the art review identified numerous different QOL measures [3]. Most QOL measures ask individuals to assess their QOL using predetermined items. This is true for both generic measures such as the Medical Outcome Study Short Form (SF-36) [4], and disease specific measures such as the Diabetes Quality of Life for Youth questionnaire (DQOLY) [5,6] and the Audit of Diabetes Dependent Quality of Life (ADDQoL) [7]. A more recent approach to QOL assessment is the development of individualized, or patient generated, measures that use an open ended question format. These measures allow respondents, from their own perspective, to identify the life domains that contribute most to their overall quality of life. This complimentary approach allows respondents to paint a fuller picture of their quality of life focusing on the domains that they consider important. The ADDQoL approaches this technique, in that it allows patients to indicate items on the measure that are 'not applicable' to their quality of life and weights remaining responses appropriately. Its paper and pencil format allow for its wider use than an interview format. However, it does not allow respondents to generate their own domains.

By pre-selecting life domains, and/or limiting items to those that are diabetes relevant, we limit our breadth and depth of understanding of youth with diabetes. While diabetes impacts nearly all aspects of a young person's life, it may not be the central, organizing construct under which all other domains fall. If a child with diabetes was asked "What is important to you?" and "How is that important thing going for you right now?" diabetes may or may not be mentioned. For example, in a sample of adolescents with type 1 diabetes, fewer than 1/3 of participants ranked diabetes as the most important life domain, the remainder rated it much lower [8]. And when diabetes is considered important, the rationale for its importance may vary between respondents or by developmental stage. Furthermore, we know very little about the developmental aspect of QOL. Do QOL domains differ by age? Does the importance attributed to the domains differ? Does the rationale for their importance differ?

These questions are not of solely theoretical interest. On an individual patient basis, such information may be clinically useful. Understanding the QOL domains that are important to an individual patient gives a provider information about how to motivate that individual for improved self-care. This is true for diabetes-related life domains and those domains that are not directly diabetes-related. For example, a provider treating a child who values athletics could use this information to motivate the child to improve diabetes self-care by demonstrating to him that improved glycemia could enhance his athletic performance. Using a patient's own value system to promote healthy behavior has been advocated by others [9] and is consistent with a patient centered approach to medical care. But, the tools to assess the life domains of importance for a given individual are required for such an approach. Walker and Bradley [8] found that diabetes nurse specialists who had considerable knowledge and experience of individual patients, were unable to predict accurately patients' ratings of their own QOL, nor the relative importance of the domains that constitute it, because of the subjective and complex nature of QOL.

The Schedule for the Evaluation of Individual Quality of Life-Direct Weight (SEIQoL-DW) is a theory based [10] structured interview during which respondents nominate 5 life domains that are most important to their own QOL [11]. The SEIQoL-DW has been used with a variety of adult and geriatric medical populations including patients with HIV/AIDS [12], cancer [13], amyotrophic lateral sclerosis [14], psychiatric diagnoses [15], multiple sclerosis [16], motor neuron disease [17], Hodgkins lymphoma [18], and stem cell recipients [19]. In the only other study using the SEIQoL in youth with diabetes, Walker and Bradley [8] administered it to 15 adolescents with type 1 diabetes. What is reported here is a descriptive, exploratory study of individual quality of life in young people with diabetes. It is not the intention to advocate the use of the SEIQoL-DW in place of standard measures such as the DQOLY. Rather, the SEIQoL-DW interview was used to provide a window into the lives children and young people with diabetes beyond what is typically assessed with paper and pencil measures. Such an understanding helps us view the patient as a whole person, and approach them from their own unique perspective. Previously, we reported the appropriateness of using the SEIQoL-DW in the children from this sample under age 18 years [20]. This article reports age related differences in individual quality of life domains in an expanded sample that includes college-aged respondents.

## Methods

### Sample

Participants were campers, and young counselors who had previously been campers, at an overnight summer camp for children with diabetes. The camp serves children with diabetes in northern New England. One hundred and twenty campers from 8–15 years old attend a 2-week session. The majority of the staff has diabetes.

### Measures

#### Demographic and disease variables

For campers, parents completed a survey of demographic and disease variables for themselves, their family, and their child with diabetes. Counselors who participated in the study completed the survey of demographic and disease variables themselves. Demographic variables included family structure, socioeconomic status, school performance, and race/ethnicity. Disease variables included disease duration, treatment regimen, HbA1c (gold standard measure of glycemic control), frequency of complications, and emergent use of health care services.

#### Schedule for the Evaluation of Individual Quality of Life-Direct Weighting

As described by Browne et al [19], there are three stages to administration of the SEIQoL-DW. In the first stage respondents nominate five life domains that they consider most important to their overall quality of life. Instructions were modified only slightly for youth, with an emphasis on making the language easy to understand and examples age appropriate. Participants were asked "For each of us, happiness and satisfaction in life depends on the areas of life which are important to us. When these important areas are going well, we are happy, but when they are going badly, we feel worried or unhappy. What is considered important varies from person to person. What is most important to you may not be so important to me or to your parents or friends and vice versa. I am interested in knowing what the most important areas of your life are at the moment. What are the five most important areas of your life at present – the things which make your life happy or sad at the moment?" If participants are unable to volunteer domains, examples are read from a standard list that is included in the SEIQoL-DW administration manual, and responses are noted as such. To assess a child's understanding of these directions, each participant was asked to "retell" the directions to the interviewer. If the child was unable to repeat the directions in basic terms and show understanding, they were excluded from the study. The appropriateness of the SEIQoL-DW in youth has been described elsewhere [20]. The authors took great care to ensure that those who were included yielded valid data, and those who did not were excluded.

In the second stage, the respondents rate each domain on a 0–100 mm vertical visual analogue scale anchored at the two extremes by the terms 'best possible' and 'worst possible'. These anchors are designed to allow individuals to use their own criteria when assessing their status within each domain.

**Figure 1:**
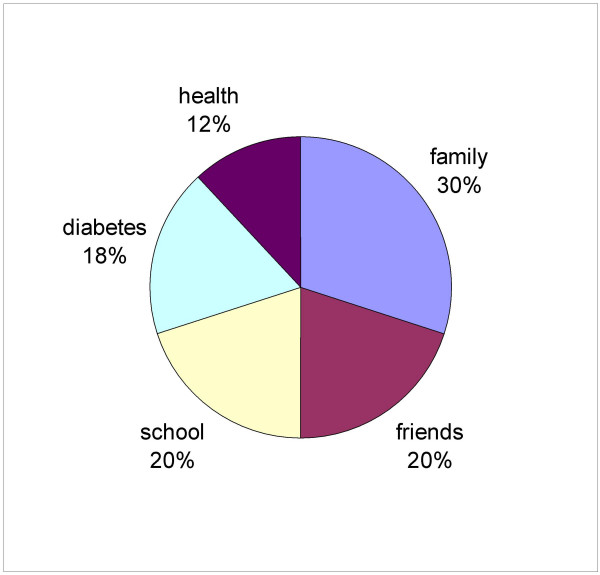
A representative distribution of life domains on the weighting instrument.

The third stage involves a weighting procedure wherein the respondent judges the relative importance of each domain. In the original version of the SEIQoL a technique known as judgment analysis was used. Because of practical limitations of judgment analysis, a direct weighting procedure has been developed [21]. The direct weighting (DW) procedure of the SEIQoL-DW consists of asking participants to manipulate five stacked, centrally mounted, interlocking laminated disks. Each disk is a different color and is labeled with one of the five domains nominated by the individual. The disks can be rotated over each other to produce a dynamic pie chart where the relative size of each sector represents the weight the respondent attaches to a QOL domain. The proportion of the chart that each sector represents can be scored from a 100-point scale on the circumference. See figure 1 for example of the weighting instrument. Total quality of life is then calculated by multiplying each domain importance rating by the domain weighting and then summing the products. Examples of this scoring have been previously published [20].

Studies have shown the SEIQoL to have good internal consistency, ranging from .6 to .9 [21-26]. It has also been shown to have adequate test-retest reliability, with Pearson's correlation >.70 [27]. The newer SEIQoL-DW has been shown to be sensitive to change, to have good construct validity [27,28], and to be psychometrically comparable to the SEIQoL [21,26]. A recent review concluded that the SEIQoL is superior to other patient generated QOL measures [29].

### Procedures

This project was carried out in accordance with the American Psychological Association guidelines for ethical conduct of research. It was approved by the University of Connecticut Health Center's institutional review board. One week prior to the two-week camp session, a letter was sent to the parents of campers, describing the study. Parents were sent a consent form for themselves, an assent form for their child, and a survey of disease and demographic data. Upon their arrival at camp, the materials were collected from parents and reviewed for completeness. Sixty one percent (n = 73) of campers and parents handed in completed questionnaires on the first day of camp. The most common reason given for not participating was lack of interest on the part of the child. During the 2-week camp session children were pulled one at a time from regular camp activities and administered the SEIQOL-DW. Participants were allowed to choose a sugar free treat (soda, gum, or mints) for their participation.

During the one-week of pre-camp (when counselors are preparing for arrival of campers), counselors with diabetes who had previously been campers were approached and invited to participate in the study. Informed consent was obtained, and staff members were administered the SEIQoL-DW individually at their convenience. All eligible staff chose to participate (n = 17).

Two interviewers completed all 90 interviews. They were the first author who is a clinical psychologist, and the third author who is a medical student with an M.P.H. in health behavior. Both interviewers studied the SEIQoL-DW manual, role-played giving the SEIQoL-DW, and conferred about validity, boredom, and fatigue scoring.

## Results

### Participants

Consenters were compared to the total camp population for systematic differences. Results of a chi square test show no significant differences in gender. Results of an ANOVA show no significant differences in age, HbA1c, or duration of diabetes. Likewise, there were no significant differences between campers and counselors for quality of life total scores, gender, type of diabetes, age of diagnosis, number of daily injections, hypoglycemic episodes in the previous month, as well as number of sick days, hospitalizations, ketoacidosis episodes, and emergency room visits in the previous year.

On average, participants were 13 years old, came from 2-adult homes (74%) with 1 or 2 siblings. All but one participant were European American. Most did "well" or "very well" in school (69%), and had parents with at least 2 years education beyond high school. Participants had diabetes for an average of about 6 years and had been attending diabetes camp for an average of 4 years. All were on multiple injection regimens or insulin pumps (n = 29), and their average glycemic control was fair (mean HbA1c = 8.02). Participants had missed an average of 3 days of school in the last year due to their diabetes, and had experienced about 6 episodes of hypoglycemia in the previous month.

**Table 1:** Demographic and diabetes descriptive findings, n = 80 (numerical discrepancies reflect missing values)

	Mean (SD)
Sex
Male	47.5% (n = 38)
Female	52.5% (n = 42)
Age	14.0 (3.4)
Age at diagnosis	7.1 (3.1)
Years since diagnosis	7.0 (4.3)
Most recent HbA1c	8.1 (1.8)
# Injections/day	2.2 (2.1)
# Children on CSII (n)	29
Diabetes sick days from school in last year	3.3 (5.4)
Diabetes hospitalizations in last year	
0	79.5 % (n = 61)
1–2	18.2% (n = 15)
>2	2.2% (n = 2)
DKA episodes in last year	
0	72.9% (n = 53)
1–2	15.3% (n = 12)
>2	11% (n = 10)
Hypoglycemic episodes in last month	6.2 (6.5)
Years at diabetes camp	4.6 (3.6)
# of siblings	1.8 (1.4)
Parent education (in years)	14.4 (2.6)
Parent marital status	
Single/separated/divorced & living alone	16% (n = 12)
Single/separated/divorced & cohabitating	17% (n = 14)
Married	66% (n = 52)
School performance	
Very poorly	1% (n = 1)
Poorly	4% (n = 4)
Ok	26% (n = 19)
Well	33% (n = 26)
Very well	36% (n = 30)

Data from ten children were deemed invalid due to interviewers' judgment that the participant was unable to understand the SEIQoL-DW task. For example, one child did not understand the concept of 'importance', and instead only rated domains in terms of his happiness with them. All children whose data were deemed invalid were under 12 years old, with a mean age of 9.25. The data from these ten children (1/3 of those children under 12) are deleted from the following results. Table 1 presents these descriptive findings.

### Domain nomination

Of the 400 total domains nominated by the 80 participants with valid data, only 21 domains (5%) were nominated with the assistance of the standard list. These 21 were for the third, fourth, and fifth domains. Thus, every respondent could nominate at least 2 domains without suggestion, and only a handful needed help with the additional 3 domains. Table 2 displays the domains and the frequency with which they were nominated.

**Table 2:** SEIQoL-DW domains, with importance and satisfaction ratings

Domain	Number of times domain was nominated	Mean importance rating out of 100	Mean satisfaction rating out of 100
Family	76	27.9	79.8
Friends	62	18.0	76.8
Diabetes	49	27.7	75.0
School	46	17.7	65.5
Health	30	22.9	80.1
Hobbies	26	15.3	66.2
Sports	17	14.2	67.6
Camp	14	15.5	89.9
Religion	12	30.6	83.8
Special person, such as a teacher	11	19.8	79.8
Approach to life, or mental attitude	10	20.0	69.6
Significant other (boyfriend/girlfriend)	9	19.0	62.5
Golden rule (treating others as would like to be treated)	9	20.1	64.7
The basics (housing, food, safety)	9	17.8	71.5
Career/future	7	15.5	62.8
Pet	7	18.3	82.1
Work	5	11.5	69.5
Nature	1	14.5	70.0

The most frequently nominated domain was 'family'. Family was nominated by 76/80 respondents. Answers were coded 'family' if the participant used the terms 'family' or 'parents'. Reasons given tended to involve either instrumental support (e.g., they provide me with clothes and a place to live) or emotional support (they love me and I can go to them with problems). Many respondents stated that their families were important because they helped with diabetes, e.g., purchased diabetes supplies, drove them to doctor's appointments, cooked nutritious food, and helped with treatment decisions. Results of a bivariate correlation indicate a significant negative correlation between age of respondent and the importance rating given to the 'family' domain, r = -.34, *p < .01. There was no relationship between respondent age and 'family' satisfaction.

The second most frequently nominated domain was 'friends'. Respondents tended to refer to a group of friends, rather than an individual friend. Friends were generally valued for their emotional support, their companionship, and the participant's ability to relax and have fun with them. The ability to "be myself" and still be accepted by friends was a common theme. Results of a bivariate correlation indicate a significant positive correlation between age of respondent and the importance rating given to the 'friends' domain, r = .35, *p < .01. There was no relationship between respondent age and 'friends' satisfaction.

School was nominated by many respondents as an important life domain. School was deemed important for a variety of reasons. Some respondents stated that school was important because learning is important in its own right. Some stated that school performance was important to assure acceptance to a good college and have a good job, or because their school performance was important to their parents. Age was not related to 'school' importance or satisfaction.

The nomination of 'diabetes' as an important life domain was common. However, respondents' explanations for its nomination were varied. One type of response referred to taking proper care of diabetes in order to avoid negative consequences. These responses included things like eating well, self-monitoring blood glucose, keeping active, and taking injections on time to avoid medical complications, hospitalization, or death. Respondents who gave this sort of answer were fairly concrete and said things such as "I have to take my shots or I will end up in the hospital". Thirty four responses fell into this diabetes 'self-care behaviors' category. Another type of diabetes response referred to living well with diabetes. These responses included things like doing enjoyable activities despite diabetes, successfully negotiating diabetes treatment with parents, feeling proud of self when diabetes is controlled, receiving emotional support for diabetes, and keeping self, friends and family from worrying about diabetes. Respondents who gave this sort of answer provided more abstract explanations, and said things such as "I can't let diabetes stop me" and "It's important that people around me understand what it's like for me to have diabetes". Eleven responses were of this type. Results of an ANOVA with rationale for diabetes nomination (self care vs. living well with diabetes) as the independent variable, and age as the dependent variable revealed that those who provided 'self care behaviors' as a rationale were younger (M = 11.8 years) were than those who provided living well with diabetes as a rationale (M = 14.6 years) F (3, 40) = 2.88, p < .05. There were no group differences for age of diagnosis, number of daily injections, hypoglycemic episodes in the previous month, as well as number of sick days, hospitalizations, ketoacidosis episodes, and emergency room visits in the previous year. Among those who nominated 'diabetes' as a domain, age was not related to 'diabetes' satisfaction.

Respondents who nominated 'diabetes' as a domain were asked whether or not they would do so if they were in a non-diabetic environment such as school, as opposed to a diabetes summer camp. All respondents stated yes, they would nominate diabetes. However, some went on to say that they might not use the word 'diabetes' per se, and instead might use the word 'health'. Twenty four respondents did in fact nominate 'health' as a domain (but not diabetes). Explanations for 'health' focused on what might be considered more general wellness. Individuals who nominated 'health' said things like "I have to be healthy in order to do the things I enjoy", "I like staying fit", "you can't be happy without good health", and "when I don't feel well I'm in a bad mood". Six other respondents nominated both 'diabetes' and 'health' as separate domains. Results of a one-way ANOVA reveal that respondents who nominated 'diabetes' only were significantly younger (M = 12.9 years) than respondents who nominated 'health' only (M = 15.9 years) F (3, 75) = 4.53, p < .01. There were no group differences for age of diagnosis, number of daily injections, hypoglycemic episodes in the previous month, as well as number of sick days, hospitalizations, ketoacidosis episodes, and emergency room visits in the previous year. Among those who nominated 'health' as a domain, age was not related to 'health' satisfaction.

### Domains nominated that are not on the standard list

Many domains were nominated by this sample that are not included in the standard list. They include 'diabetes', 'school', and 'camp', all of which clearly reflect that this was a sample of school aged young people attending a camp for children with diabetes. Some respondents nominated a 'special person' in their lives, such as a teacher or a coach. Other novel domains were nominated the explanations for which are less obvious. One domain involved 'mental attitude' and referred to taking the right approach to life and maintaining a positive outlook. Another involved 'the golden rule' and referred to treating others as one wants to be treated, with fairness and respect.

As stated above, the most frequently nominated domains were 'family', 'friends', 'diabetes', 'school', and 'health'. Domains ranked by importance were 'religion', 'family', 'diabetes', 'health', and 'the golden rule'. Domains ranked by satisfaction were 'camp', 'religion', 'pets', 'health', and 'family' and 'a special person' tied for fifth.

### Quality of Life

Total SEIQoL-DW scores ranged from 34.9 – 97.7, M = 78.1, SD = 11.2. Normative data for children are not available. However, these values are similar to those of a small clinic sample of adolescents with type 1 diabetes that reported SEIQoL satisfaction data only [8]. This and other clinical samples (adults with cancer, with mental illness, transplant recipients) are shown in Table 3.

**Table 3:** Means and SDs for the SEIQoL-DW for the study and comparison samples

Sample	Mean	SD
Youth with diabetes	78.1	11.2
Adolescents with diabetes (satisfaction ratings only) [8]	75.3	N/A
Adult HIV/AIDS patients [12]	58.4	21.59
Adult community serious mental illness [15]	69.04	24.58
Adult advanced cancer patients [26]	50.9	17.8
Adult stem cell transplant recipients [19]	63.21	17.55

## Discussion

The purpose of this study was to explore the individual QOL domains of youth with diabetes. Consistent with previous findings [8] results indicate that life domains nominated by individuals were thematic and shared many common characteristics, but varied substantially across respondents. Moreover, even when different respondents nominated identical life domains, their rationales for the importance of those domains varied. These findings underscore the personal nature of QOL, and highlight the benefit of allowing individuals to express their views of the life domains that determine it.

The young people in this sample nominated nearly all the life domains that are offered on the SEIQoL-DW standard list (with the exception of finances). They also went on to nominate additional domains, notably what we have termed 'mental attitude' and 'the golden rule'. These domains are some of the more fundamental aspects of quality of life but are not typically nominated in adult samples. Their nomination raises interesting questions. It is possible that youth are more concerned with these life domains than adults. This may be particularly true for children at overnight summer camp where community living and group cooperation is fundamental and continually reinforced. It is also possible that people with diabetes are more concerned with these life domains than their non-diabetic counterparts. Perhaps these respondents, who have experienced a major negative life event with their diabetes diagnosis and may also have suffered teasing or rejection due to this diagnosis, are more sensitive to issues of positive thinking and treating others with respect. Because this was an uncontrolled study, these are, of course, hypotheses that can only be tested with further investigation of controls.

Nearly all respondents nominated a domain that reflected physical status. Younger respondents (who were on average 12 years old) were more likely to focus specifically on diabetes, and emphasize the importance of diabetes self-care behaviors. These respondents stressed adherence to the diabetes treatment regimen in order to avoid medical complications. Older respondents (who were on average 15 years old) were more likely to focus on general health and emphasize the need to live well despite the difficulties of diabetes. These age related data need to be interpreted cautiously due to small sample size, homogeneity of the respondents, and the cross sectional design of the study. The literature would benefit from further use of individual QOL measures with children and with chronic illness populations such as diabetes, in longitudinal designs. Nonetheless, these data do suggest a change in how diabetes is conceptualized during adolescence.

There are several possible explanations for these age differences. First, Piaget proposed that formal cognitive operations begin in adolescence. With formal operations, adolescents gain the ability to think about their own thinking, to imagine many possibilities, and to mentally generate possible outcomes and thus rely less on real objects and events. Abstract thought becomes possible. The observed shift in focus from the concrete to the abstract – from 'diabetes' to 'health' and from 'self-care behaviors' to 'living well' – may be a reflection of the cognitive development that occurs with formal operations. Studies have demonstrated a systematic progression of children's understanding of illness that corresponds to Piaget's framework [30,31]. Other research has shown that the growth of children's conceptualization of illness paralleled, but lagged behind, conceptual development of physical causality [32]. Indeed, cognitive development may explain some children under 12 were not able to comprehend the SEIQoL-DW instructions.

Second, as they go through adolescence, youth with diabetes may view their physical well being differently due to more extensive and broadened life experience. Older youth may realize that strict adherence to the medical regimen comes at a cost of an inflexible lifestyle, and may value quality of life over strict medical management. They may become more willing to make compromises in self-care in order to participate more fully in normal activities. They may also, for the first time, be in a position to make these compromises as parental control of day-to-day diabetes management wanes in adolescence. Third, this shift may reflect adolescents desire to fit in with peers, normalize their disease experience, and assimilate their illness. Viewing physical status in terms of health, rather than diabetes, and diabetes in terms of living well, rather than disease management behaviors, serves these functions.

This shift in diabetes conceptualization could serve to be adaptive, or conversely it may herald the poor self-care and decreased glycemic control that is often noted in adolescence and young adulthood. Wysocki, Hough, Ward and Green [33] found that adolescents with diabetes are at risk of various unfavorable behavioral and health outcomes and that adjustment to the disease during earlier adolescence may be a predictor of subsequent health-related behavior and health status. We did not find a relationship between glycemic control and specific life domains nominated; this could be a result of low statistical power or a true lack of relationship. Also, we did not measure psychological adjustment to diabetes, and it remains an empirical question whether the conceptual shift observed in the present study is related to adjustment, and if so, the direction of the relationship. Furthermore, this shift in itself may be less important than host variables such as health beliefs and social support. Perhaps for a well adjusted, well supported individual, the shift toward general health may indicate disease assimilation, where as in less adjusted, less supported adolescents, the shift away from diabetes may indicate a denial of the disease and a withdrawal from appropriate self-care.

In addition to differences in domain nomination, we also observed age effects for the importance assigned to domains. As would be expected, age was significantly positively associated with the reported importance of friends, and a significantly negatively associated with the reported importance of family. As young people grow older, the value they place on family and friends changes, and peers become increasingly important. These data are consistent with previous findings that children with diabetes find friends more helpful in diabetes management than many adults [34]. Interventions that include peers, or a 'diabetes buddy' may be complimentary to those that target the family. A clinician who knows what a patient values can use that information to build rapport and to promote healthy behaviors. For example, while only 12 respondents nominated religion as a domain, it was given a higher importance rating than any other domain. Asking "how would God want you to care for your diabetes?" may be more fruitful with these children than a discussion of, say, the benefits of glycemic control for sports performance.

These data should be interpreted cautiously for several reasons. First, they reflect a select sample of white, higher socioeconomic status, high academic achieving children, many of whom have had several years of diabetes camp experience. This group may nominate different life domains, and endorse a higher QOL, than children who come from more impoverished environments without disease specific psychosocial experiences. These findings should be viewed tentatively until findings are replicated with larger and more diverse samples, in longitudinal designs that employ controls.

## Conclusions

This article reports age-related differences in health related quality of life domains in youth with type 1 diabetes. It was found that younger respondents nominated 'diabetes' as a domain and focused on 'self-care behaviors', whereas older respondents nominated 'health' and focused more on 'living well with diabetes'. Although limited by sample size and homogeneity, these findings point to age related differences that may help explain deteriorating glycemic control during adolescence. What is clear is that development into maturity is a difficult task made more difficult by diabetes, and that special attention must be given to this population. The Society for Adolescent Medicine [35] and the American Academy of Pediatrics [36] have both issued statements stressing the importance of transitional care from pediatrics to adult medicine for youth with special health care needs. Grey et al. [37] conclude that diabetes treatment teams need to pay attention to the psychosocial needs of all adolescent patients. As the medical management of youth with diabetes improves, so must the understanding of behavioral and psychosocial status. These data shed light on the quality of life domains valued by youth with diabetes.

## Authors' contributions

JW conceived of the study, and was responsible for design, analysis, and manuscript preparation. GA assisted with data analysis, and manuscript preparation and revision. SL carried out many of the interviews, assisted with data recording, data entry, and study coordination.
